# Studies on Molecular Characterizations of the Outer Membrane Proteins, Lipids Profile, and Exopolysaccharides of Antibiotic Resistant Strain *Pseudomonas aeruginosa*

**DOI:** 10.1155/2015/651464

**Published:** 2015-02-01

**Authors:** Hany M. Yehia, Wesam A. Hassanein, Shimaa M. Ibraheim

**Affiliations:** ^1^Food Science and Nutrition Department, College of Food and Agriculture Sciences, King Saud University, Saudi Arabia; ^2^Food Science and Nutrition, Faculty of Home Economics, Helwan University, Egypt; ^3^Department of Botany (Microbiology), Faculty of Science, Zagazig University, Zagazig, Egypt

## Abstract

Susceptibility of the tested *Pseudomonas aeruginosa* strain to two different antibiotics, tetracycline (TE) and ciprofloxacin (CIP), was carried out using liquid dilution method. Minimum inhibitory concentrations of TE and CIP were 9.0 and 6.0 mg/100 mL, respectively. Some metabolic changes due to both, the mode of action of TE and CIP on *P. aeruginosa* and its resistance to high concentrations of antibiotics (sub-MIC) were detected. The total cellular protein contents decreased after antibiotic treatment, while outer membrane protein (OMP) contents were approximately constant for both treated and untreated cells. Sodium dodecyl sulphate polyacrylamide gel electrophoresis (SDS-PAGE) analysis of the OMPs for untreated and TE and CIP treated cells indicated that the molecular changes were achieved as; lost in, induction and stability of some protein bands as a result of antibiotics treatment. Five bands (with mol. wt. 71.75, 54.8, 31.72, 28.63, and 20.33 KDa) were stable in both treated and untreated tested strains, while two bands (with mol. wt. 194.8 and 118.3 KDa) were induced and the lost of only one band (with mol. wt. 142.5 KDa) after antibiotics treatment. On the other hand, total lipids and phospholipids increased in antibiotic treated cells, while neutral lipids decreased. Also, there was observable stability in the number of fatty acids in untreated and treated cells (11 fatty acids). The unsaturation index was decreased to 56% and 17.6% in both TE and CIP treatments, respectively. The produced amount of EPSs in untreated cultures of *P. aeruginosa* was relatively higher than in treated cultures with sub-MICs of TE and CIP antibiotics. It was also observed that the amounts of exopolysaccharides (EPSs) increased by increasing the incubation period up to five days of incubation in case of untreated and antibiotic treated cultures.

## 1. Introduction

Bacterial cells grow and divide, replicating repeatedly to reach the large numbers present during an infection or on the surface of the body. To grow and divide, organism must synthesize or take up many types of surrounding biomolecules. Antibiotics interfere with the specific process that is essential for this growth and/or division of bacteria [1].

There were four major sites in bacterial cell that they serve as the basis for the action of antibiotics: the cell wall, ribosome, nucleic acids, and cell membrane [2]. This classification did not mean that antibiotics inhibited only the mentioned vital process of the bacterial cell; each particular antibiotic was classified by the mechanism of its concentration, chemical nature, or other microbial conditions [3].

On the other hand, the basic mechanisms by which microorganisms can resist antibiotics were (1) to alter the receptor for the drug (the molecule on which it exerted its effect) as fluoroquinolones, (2) to decrease the amount of drug that reached the receptor by altering entering or increasing removal of the drug as tetracyclines, (3) to destroy or inactivate the drug, (4) to develop resistant metabolic pathways, [1] and (5) to exclude from the cell by the outer membrane of Gram-negative bacteria [4].

The relation between resistances of Gram-negative to different antibiotics, the outer membrane proteins, and lipids composition of these organisms was provided. Hydrophilic antibacterial agents were prevented from entering through the outer membrane by the lipopolysaccharide layer and the underlying phospholipids, whereas hydrophobic agents were excluded by outer membrane protein. Hypersusceptibility to antibiotics might occur when the lipopolysaccharide had been altered but also when the outer membrane proteins had remained constant [5].

On the other hand, exopolysaccharides were produced by many bacteria from clinical and environmental habitats [6]. Production of EPSs by* Pseudomonas* species had been widely reported [7].

Exopolysaccharides were believed to protect bacterial cells; EPSs matrix provided an effective barrier that restricted penetration of chemically reactive bioacids, cationic antibiotics, and antimicrobial agents; for that, these EPSs played an important role in bacterial resistance by imitating diffusion of antibiotics to cells [8]. Also, they protect bacterial cells from desiccation, heavy metals, organic compounds, or other environmental stresses [9].

EPSs produced by certain important bacteria such as* P. aeruginosa, P. fluorescens, P. stutzeri*, and* P. putida* had a potential interest in biotechnological applications [10].

This research aimed to study the growth pattern of the tested* P. aeruginosa* strain treated with tetracycline and ciprofloxacin separately. Investigation of the role of outer membrane proteins, lipid fractions, fatty acids, and exopolysaccharides in the resistance of this strain to tested antibiotics.

## 2. Materials and Methods

### 2.1. Bacterial Strain

*Pseudomonas aeruginosa* strain was kindly provided from Microbiological Laboratory of General Surgery Department, Zagazig University Hospitals.

#### 2.1.1. Antibiotic Treatment

Two antibiotics were used: tetracycline TE capsule (500 mg) (Chemical Industry Development Co., Egypt) and ciprofloxacin CIP suspension (200 mg) (Amirya Pharmaceutical Industries, Egypt). Using liquid dilution method, different concentrations of each antibiotic were supplemented to 100 mL King's B broth medium, pH 7 ± 0.2. Each treatment was inoculated with 1 mL of bacterial suspension for 24 hr and at age 0.05 McFarland. Cultures were incubated for 24 hr at 37°C. The growth was determined by measuring turbidity of liquid cultures at 600 nm using spectrophotometer. The minimum inhibitory concentration was determined in liquid cultures visually as the lowest concentration of antibiotic which prevent the visible growth [11].

### 2.2. Protein Analysis

#### 2.2.1. Determination of Total Protein Contents

The protein contents (as mg/mL) of both untreated and antibiotic treated liquid bacterial cultures were determined using the modified assay of Lowry et al. [12]. Sub-MIC of TE (8.0 mg/100 mL) and CIP (5.0 mg/100 mL) were used separately for various studies.

### 2.3. Outer Membrane Proteins Pattern

OMPs of both untreated and antibiotic treated (at sub-MIC) strains were extracted by the method [13] with minor modification. Bacteria were grown for 48°C at 37°C in 100 mL of King's B broth. Cells were recovered by centrifugation (6,000 ×g for 10 min at 4°C), suspended in 3 mL of HEPES (N-2-hydroxyethylpiperazine-N′-2-ethanesulfonic acid, Sigma Chemical Co., St. Louis, MO, 10 mM, pH 7.4), and disrupted by sonication (Braunsonic sonifier, 45 s at 50% output). Cell debris was removed by centrifugation at 6,000 ×g for 10 min at 4°C. The supernatant was added to 0.75 mL of 2% N-lauroylsarcosine (Sarkosyl, Sigma Chemical Co.) and incubated for 10 min at room temperature. The mixture was centrifuged at 100,000 ×g for 1 hr (Beckman 70.1 Ti, 39,000 rpm) in order to recover the detergent-solubilized OMPs. The pelleted proteins were resuspended in 3 mL of 10 mM HEPES (pH 7.4), incubated with 1 volume of sarkosyl at room temperature for 20 min, and recovered by ultracentrifugation as described above. The final pellet was resuspended in 1 mL of 10 mM HEPES and stored at −20°C. Sodium dodecyl sulfate-polyacrylamide gel electrophoresis (SDS-PAGE) [14] was carried out with a 4% stacking and a 90% separating gel after the OMP preparations were solubilized at 100°C for 7 min in 0.05 M Tris-HC1 buffer (2.5% SDS, 5% 2-mercaptoethanol, 25% glycerol, and 0.003% bromophenol blue). Major protein bands were visualized with silver stain (silver stain-Daiichi kit, Integrated Separation Systems, Hyde Park, MA). On the other hand, protein content of OMP was also determined as mg/mL using method [12].

### 2.4. Lipid Analysis

#### 2.4.1. Preparation of Bacterial Dry Weight for Analysis

Antibiotic treated (8.0 or 5.0 mg/100 mL TE and CIP, resp.) and untreated bacterial liquid cultures (500 mL) were centrifuged for 15 min at 5000 rpm, washed with distilled water, transferred to bottles with known weight, and then dried at 60°C up to constant weight. The dry weights were subjected to lipid analysis.

#### 2.4.2. Total Lipids Extraction

Total lipids of treated and untreated cells were extracted according to the method [15].

Total lipid contents of known weight of bacterial dry cells were extracted with mixture of chloroform/methanol (2 : 1 V/V) for 24 hr. The residues were reextracted again with chloroform/methanol mixture for 2 hr and evaporated under vacuum condition. This crude lipid residue was resuspended in chloroform and washed with sodium chloride solution (0.9%) in separating funnel, then dried by passing through solid anhydrous sodium sulfate, transferred to a known weight container, and evaporated at room temperature to get rid of the chloroform. Total lipids were calculated as % of dry weight. The total lipid extract was dissolved in known volume of chloroform (5 mL) and stored at −5°C in glass Stoppard volumetric flask.

#### 2.4.3. Estimation of Total Phospholipids

Phospholipids were determined according to the method described by [16]. Known volume of the total lipids extract (50 *μ*L) was digested with 0.4 mL of perchloric acid by heating on direct flame until the digest was clear. The digest was cooled and diluted with 4.2 mL distilled water. 0.2 mL of ammonium molybdate solution (5%) followed by 0.2 mL of amidol solution (1% amidol in 20% sodium metabisulfite) was added. The tubes were then transferred to boiling water bath for 7 min and cooled with cold water. After 15 min optical density of the stable blue color was measured at 830 nm. Standard phosphate solution KH_2_PO_4_ (0.445 g/100 mL H_2_O) was prepared as stock solution which was then diluted 100 times with distilled water to give 10 ug/mL. Standard phosphate serial dilutions (1–10 *μ*g) were prepared. The blank was prepared using 1 mL of distilled water. Total phospholipids were calculated as % of total lipids.

#### 2.4.4. Estimation of Fatty Acids

Analysis of fatty acids (as methyl esters) was conducted according to method [17] by gas chromatography. Total lipid samples were methylated to fatty acid methyl esters by adding two mills of borontrifloride in methanol. The tubes were boiled for 2-3 min in a water bath and then cooled in ice and 2 mL of distilled water was added. A suitable volume of hexane was added; the hexane layer which contained methyl ester was separated by using separating funnel and then evaporated. The residue was suspended in known volume of chloroform for injection in Hewlett Packard gas chromatograph model 5890 located at Microbiology Laboratory of Water and Land Reclamation Unit, Agriculture Research Center, Giza, Egypt. Fatty acids methyl esters were identified by comparing their retention time with those of authentic methyl esters standards (Sigma Co., USA). The relative amount of each fatty acid of methyl esters was calculated from the integrated area of each peak and expressed as a percentage of the total area of all peaks.

### 2.5. Determination of Total Exopolysaccharides (EPSs)

Total exopolysaccharides of antibiotic treated (8.0 or 5.0 mg/100 mL TE and CIP resp.) and untreated* P. aeruginosa* strain were determined according to the method [18]. Bacteria were grown in King's B broth medium overnight at 37°C and centrifuged at 15000 rpm for 15 min. The pellets obtained were suspended in 5.0 mL of 0.85% KCl solution and inoculated into 250 mL serum bottle containing 100 mL culture medium. One hundred mL batch cultures were incubated at 28°C for 1–5 days with constant agitation in an orbital shaker (150 rpm). The cultures were centrifuged at 18000 rpm. The pH of the supernatant was then adjusted to 7.2; polysaccharides were precipitated by the addition of 6.0 gram of NaCl, followed by adding equal volume of 95% ethanol. The precipitate was recovered by centrifugation at 3000 rpm for 15 min. Polysaccharides were then dehydrated in an alcohol series (60, 70, 80, and 95% ethanol) and then dried at 35°C. The weight of EPSs was determined as g/L.

#### 2.5.1. Statistical Analysis

The obtained data were statistically analyzed to determine the means, standard deviation, and one sample *t*-test as described [19]. Bivariate correlation matrix of the obtained data was done using SPSS software program (ver. 8) as described [20].

## 3. Results and Discussion

Resistance had been defined as the temporary or permanent ability of an organism and its progeny to remain viable and/or multiply under conditions that would destroy or inhibit other members of the strain. Resistance referred to instances where the basis of increased tolerance was a genetic change and where the biochemical basis was known. Antimicrobial substances target a range of cellular loci, from the cytoplasmic membrane to respiratory functions, enzymes, and the genetic material [21].

Through this research, resistance of the tested* P. aeruginosa* strain to two different antibiotics TE and CIP was examined. Tetracyclines are a group of antibiotics acting as inhibitors for protein synthesis. They bind to 30S ribosomes subunits and inhibit their function during protein process [1]. On the other hand, ciprofloxacin is fluoroquinolones which act as inhibitors of DNA replication [21].

Results in Table 1 indicated the growth pattern of the tested* P. aeruginosa* strain measured at 600 nm under increasing concentrations of TE and CIP antibiotics separately. The results indicated that the growth decreased as the concentrations of each tested antibiotic increased. Also, visible determination of the growth indicated that MICs were 9.0 and 6.0 mg/100 mL for TE and CIP, respectively. This result revealed that the tested strain was more resistant to TE than to CIP. In this connection the authors reported that three of* P. aeruginosa* out of 90 isolates were resistant to ciprofloxacin and exhibited MIC values of 16–32 mg/L [22].

In this connection, the resistance to tetracycline was normally due to the acquisition of new genes [23]. This resistance was primarily due to either energy dependent efflux of tetracycline or protection of the ribosomes from its action. Gram-negative TE efflux proteins were linked to repressor proteins which in the absence of TE block transcription of the repressor and structural efflux genes.

Antibiotics may have multiple sites of action in bacterial cell. Biochemical, cytological, molecular, biological, and genetic changes were induced as a result of antibiotic action. Through this research, changes in protein contents, OMPs pattern, lipid fractions, and EPSs of the tested* P. aeruginosa* strain due to the mode of action of both TE and CIP separately on the organism and its resistance to high concentrations (sub-MIC) were studied.

Results in Table 2 indicated the total cellular proteins and OMPs contents (as mg/mL) of untreated and antibiotic treated strain. The results indicated that although total cellular protein contents decreased by 27.4 and 37.2% after treatment with TE and CIP, respectively, compared to untreated one, the OMPs contents were approximately constant in both untreated and treated strains. The reduction of total cellular protein contents might be referred to the mode of action of antibiotic on bacterial cell, where tetracycline inhibited the binding of aminoacyl-tRNA into the A site of the bacterial 30S ribosome and consequently inhibited protein synthesis [1]. On the other hand, ciprofloxacin might inhibit one or more enzymes, such as DNA topoisomerase enzyme including DNA* gyrase*, which was essential in DNA replication. This effect caused DNA coagulation. Also thickening of the outer membrane was the action of this group of fluoroquinolones. This effect was only present when the bacteria were threatened within its replication or during synthesis of the essential protein in their growing phase [6]. Moreover, stability in the OMPs contents in treated and untreated strain explained the role of these proteins in resistance of tested strain to both TE and CIP.

From the previous experiment, it was found that it was very important to study the molecular characterization of OMPs for TE and CIP treated and untreated cells of the tested* P*.* aeruginosa* strain.

**Figure 1:**
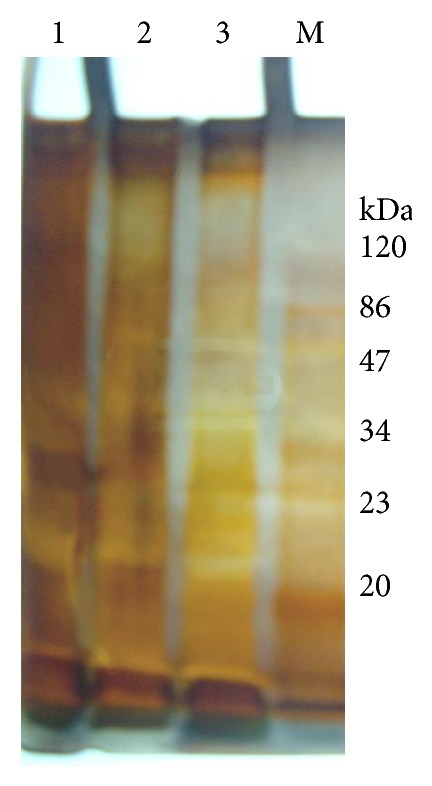
Outer membrane proteins banding pattern of antibiotics treated and untreated* P. aeruginosa* strain. M, marker; lane 1, untreated cells; lane 2, treated cells with 5.0 mg/100 mL CIP; and lane 3, treated cells with 8.0 mg/100 mL TE.

Results in Table 3 and Figure 1 indicated the molecular weight and the amount (%) of outer membrane proteins extracted from untreated and TE (8.0 mg/100 mL) and CIP (5.0 mg/100 mL) treated cells of the tested* P. aeruginosa* strain using SDS-PAGE analysis. The results showed that five bands with molecular weights 71.75, 54.8, 31.72, 28.63, and 20.33 KDa were stable in both untreated and antibiotic treated samples. Also, two bands with molecular weights 194.8 and 118.3 KDa were newly induced, while only one band with molecular weight 142.5 kDa was lost after treatment with each of TE and CIP separately. On the other hand, the results illustrated that the total amount (%) of OMPs increased in treated strain compared to untreated one and its value was approximately equal in both TE and CIP treated strains.

Lipid profile is one of the most important characterizations in Gram-negative bacteria. Bacterial lipids were classified into nine groups: hydrocarbons, glycerides, waxes, steroids, phospholipids, glycolipids, lipopolysaccharides, peptidolipids, and peptidoglycolipids [9]. Most of these compounds are localized in cell wall and also distributed in cell membrane and outer membrane [1].

Antibiotics and different antibacterial compounds had an influence upon the lipid metabolism. Through this research the effect of both TE and CIP treatments on lipid profile and fatty acids was studied.

Results in Table 4 indicated that total lipids and phospholipids increased in antibiotic treated cells of the tested* P. aeruginosa* strain. Total lipids increased by 25.7 and 10.9% after treatment with each TE and CIP, respectively, compared to untreated one. Phospholipids increased by 4.0 and 1.7% for TE and CIP treatment, respectively, compared to untreated one. On the other hand, neutral lipids decreased by 5.9 and 2.5% for TE and CIP treatment, respectively. Also the results indicated that increase in total phospholipids in TE compared to CIP treated cells might be correlated to high resistance of organism to TE more than to CIP.

In this relation, it could be concluded that the resistant mutant of bacteria to antibiotics had more total lipids and phospholipids contents than sensitive one [10, 11]. Also, there was relationship between the amounts of envelop phospholipids and sensitivity of Gram-negative bacteria to polymyxin [13].

Regarding the fatty acids, results in Table 5 indicated that there was observable stability in the number of fatty acids in both untreated and treated cells (11 fatty acids for each). Linolenic and oleic acids had the highest percentage in both treated and untreated strains. Myristic acid was acquired while behenic acid was lost after TE treatment. Both treatments of TE and CIP increased the percentages of caprylic, capric, and stearic acids while decreasing the percentages of oleic, linoleic, and eicosadienoic acids compared to untreated strain. On the other hand, the unsaturation index was decreased to 56 and 17.2% for both TE and CIP treatments, respectively, compared to untreated sample.

Related observation was recorded by Hassanein [24]. She found that palmitic acid represented the highest percentage of fatty acids detected in untreated and cephalosporin or kanamycin treated* E. coli*. Also, myristic, myristoleic, and linoleic acids were induced while linoleic and eicosadienoic acids were lost after cephalosporin and kanamycin treatment, respectively. Also, saturated %/unsaturated % of fatty acids decreased in antibiotic treated strain compared to untreated control one.

It can be concluded that antibiotic treatment at certain antibiotic concentrations led to altering and/or misreading of genetic code for fatty acids and lipid biosynthesis which led to the disappearance of some fatty acids and induction of others [25].

Also, this research extended to study the production of EPSs by the tested* P. aeruginosa* strain and their role in the resistance of this organism to antibiotic. Resistance to antimicrobial agents was the most important feature of biofilm infection. Although several mechanisms had been postulated to explain reduced susceptibility to antimicrobials in bacterial biofilms, it was becoming evident that biofilm resistance was multifactorial [26].

**Figure 2:**
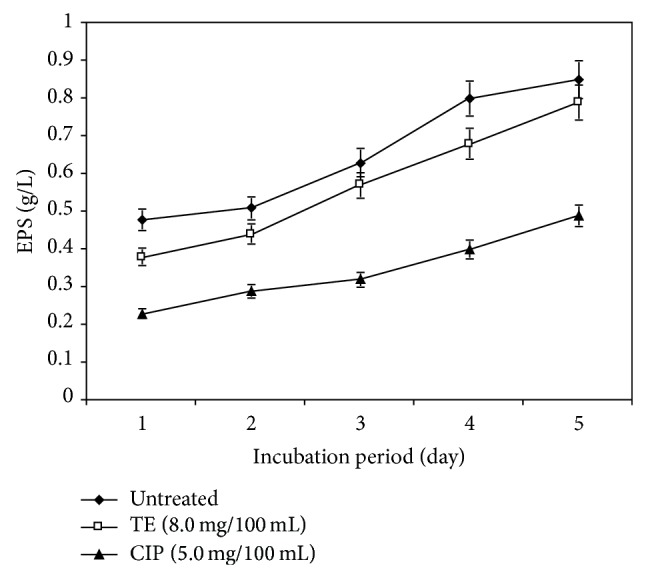
Effect of sub-MIC of TE and CIP on exopolysaccharides (EPSs) of the tested* P. aeruginosa* strain at different incubation periods.

In the light of these important facts, this research was extended to study quantitative change in the EPSs produced by the tested* P. aeruginosa* strain incubated for 5 days at sub-MICs concentrations of TE (8.0 mg/100 mL) and CIP (5.0 mg/100 mL) separately. Results in Figure 2 showed that the quantity of EPSs increased as the incubation period increased for untreated and treated samples. Also, the amount of EPSs decreased after treatment with TE or CIP compared to untreated one at all incubation periods. Moreover, the quantity of EPSs produced by* P. aeruginosa* cells treated with TE was higher than cells treated with CIP at all incubation periods. This might explain increased resistance of tested* P. aeruginosa* strain to TE compared to CIP. In this relation, Irvin et al. (1981) found that spontaneous mucoid mutants of* P. aeruginosa* 492c that grew in 50 ug/mL carbenicillin produce large amounts of EPSs when grown on the growth medium, while nonmucoid strains of 492c did not produce EPSs and were still susceptible to less than 1 ug/mL of carbenicillin. Also, Vrany et al. [27] reported that* P. aeruginosa* biofilms delayed penetration and delivery of aminoglycosides, but penetration of fluoroquinolones such as ciprofloxacin and ofloxacin occurred without delay. Hoyle and Costerton [28] suggested that the barrier to drug penetration formed by the EPSs and low growth rate of bacteria in biofilms were related to drug resistance.

## 4. Conclusion

The antibiotic treatment led to altering and/or misreading of genetic code for fatty acids and lipid biosynthesis and resulted in disappearance of some fatty acids and induction of others. The decrease in unsaturation index indicates transformation of lipids to solid state, making plasma membrane more rigid and lose its fluidity. Also, this research investigated the production of EPSs by the tested* P. aeruginosa* strain and their role in the resistance of this organism to antibiotic. In addition to the quantitative change in the EPSs produced by the tested* P. aeruginosa* strain incubated for 5 days at sub-MICs concentrations of TE (8.0 mg/100 mL) and CIP (5.0 mg/100 mL) separately, EPSs were increased as the incubation period increased for untreated and treated samples. The amount of EPSs decreased after treatment with TE or CIP compared to untreated one at all incubation periods. Moreover, the quantity of EPSs produced by* P. aeruginosa* cells treated with TE was higher than cells treated with CIP at all incubation periods. This might explain increased resistance of tested* P. aeruginosa* strain to TE compared to CIP.

## Figures and Tables

**Table 1:** Effect of increasing concentrations of TE and CIP on growth of the tested *P. aeruginosa* strain.

Antibiotic conc. mg/100 mL	OD (600 nm)	
	TE	CIP
0	0.67 ± 0.01	0.67 ± 0.01
1	0.64 ± 0.01	0.61 ± 0.01
2	0.59 ± 0.01	0.52 ± 0.01
3	0.51 ± 0.01	0.32 ± 0.01
4	0.40 ± 0.01	0.11 ± 0.01
5	0.33 ± 0.01	0.02 ± 0.01
6	0.23 ± 0.01	**MIC**
7	0.10 ± 0.01	—
8	0.03 ± 0.01	—
9	**MIC**	—
*t*	10.023	6.286
*P*	<0.001^***^	<0.001^***^

*t*: one sample test, *P*: *P* value.

^***^more highly significant at <0.001.

**Table 2:** Effect of sub-MICs of TE and CIP on total cellular proteins and outer membrane proteins contents of the tested *P. aeruginosa* strain.

Treatment	Total cellular proteins content (mg/mL)	OMPs content (mg/mL)
Untreated	11.3 ± 0.10	5.60 ± 0.10
TE (8.0 mg/100 mL)	8.20 ± 0.10	5.40 ± 0.10
CIP (5.0 mg/100 mL)	7.10 ± 0.10	5.60 ± 0.10
*t*	14.088	125.484
*P*	<0.001^***^	<0.001^***^

*t*: one sample test, *P*: *P* value.

^***^more highly significant at <0.001.

**Table 3:** Molecular weights and amount % of extracted outer membrane proteins of the tested *P. aeruginosa* strain treated with sub-MICs of TE and CIP.

Treatment	Untreated		CIP (5.0 mg/100 mL)		TE (8.0 mg/100 mL)		M	
Lanes	Lane 1		Lane 2		Lane 3		Lane 4	
Rows	Mol. wt.	Amount%	Mol. wt.	Amount%	Mol. wt.	Amount%	Mol. wt.	Amount%

r1								
r2			194.8	0.52076	194.8	1.7818		
r3	142.52	0.9757						
r4			118.3	28.639	118.3	14.699	120	29.606
r5							86	0.79774
r6					78.95	8.3631		
r7	71.75	7.3978	71.75	14.519	71.75	8.362		
r8	54.8	17.071	54.8	1.3673	54.8	7.027		
r9							47	0.6413
r10	40.663	0.56036			40.663	1.679		
r11					33.712	6.4954	34	20.721
r12	31.721	20.327	31.72	20.264	31.721	7.3197		
r13					30.135	6.2393		
r14	28.635	32.422	28.635	20.573	28.635	30.652		
r15							28	0.36329
r16	20.333	0.27476	20.333	8.2844	20.333	2.215	20	1.0261

Sum		79.03		94.168		94.8333		53.155
In lane		100		100		100		100

**Table 4:** Effect of sub-MICs of TE and CIP on lipid fractions of the tested *P. aeruginosa* strain.

Lipid fraction	Treatment		
	Untreated	TE (8.0 mg/100 mL)	CIP (5.0 mg/100 mL)
Total lipids (TL) (% of dry wt.)	10.10 ± 0.10	12.70 ± 0.10	11.20 ± 0.10
Phospholipids (PL) (as % of TL)	59.90 ± 0.10	62.30 ± 0.10	60.90 ± 0.10
Neutral lipids (NL) (as % of TL)	40.09 ± 0.01	37.70 ± 0.10	39.10 ± 0.10

*t*	5.070	5.247	5.154
*P*	<0.01^**^	<0.01^**^	<0.01^**^

*t*: one sample test, *P*: *P* value.

^**^highly significant at <0.01.

**Table 5:** Effect of sub-MICs of TE and CIP on fatty acids percentages in the tested *P. aeruginosa* strain.

Fatty acids (as % of TL)		Untreated	TE (8.0 mg/100 mL)	CIP (5.0 mg/100 mL)
(1) Caproic	C6:0	—	—	—
(2) Caprylic	C8:0	2.33	3.27	3.57
(3) Capric	C10:0	1.96	10.14	2.47
(4) Lauric	C12:0	3.03	7.09	1.23
(5) Myristic	C14:0	—	4.49	—
(6) Palmitic	C16:0	2.70	6.77	1.03
(7) Stearic	C18:0	2.53	4.24	3.23
(8) Oleic	C18:1	19.17	19.03	16.68
(9) Linoleic	C18:2	24.69	12.87	23.47
(10) Linolenic	C18:3	13.77	11.66	14.57
(11) Arachidic	C20:0	9.24	8.99	15.56
(12) Eicosadienoic	C20:1	13.99	5.98	12.66
(13) Behenic	C20:0	6.56	—	5.63

Unsaturation index		2.50	1.1	2.06
